# The increase of PTSD in front-line health care workers during the COVID-19 pandemic and the mediating role of risk perception: a one-year follow-up study

**DOI:** 10.1038/s41398-022-01953-7

**Published:** 2022-05-03

**Authors:** Hui Ouyang, Shiyu Geng, Yaoguang Zhou, Jing Wang, Jingye Zhan, Zhilei Shang, Yanpu Jia, Wenjie Yan, Yan Zhang, Xu Li, Weizhi Liu

**Affiliations:** 1 Lab for Post-traumatic Stress Disorder, Faculty of Psychology and Mental Health,, Navy Medical University, Shanghai, 200433 China; 2 The Emotion & Cognition Lab, Faculty of Psychology and Mental Health,, Navy Medical University, Shanghai, 200433 China; 3 Department of Emergency Medicine, Nanfang Hospital,, Southern Medical University, Guangzhou, 510515 China; 4 School of Economics,, Xiamen University, Fujian, 361000 China

**Keywords:** Psychiatric disorders, Human behaviour

## Abstract

The long-term health consequences of the COVID-19 pandemic on health care workers (HCWs) are largely unclear. The purpose of the present study was to investigate the development of posttraumatic stress disorder (PTSD) in HCWs in a longitudinal manner. Additionally, we further explored the role of risk perception in the evolution of PTSD over time based on a one-year follow-up study. HCWs were recruited from hospitals in Guangdong, China. Demographic information, the PTSD checklist for DSM-5 (PCL-5) and the risk perception questionnaire were obtained online at two different time points: May to June 2020 (T1), with 317 eligible responses, and June 2021 (T2), with 403 eligible responses. Seventy-four HCWs participated in the survey at both T1 and T2. The results revealed that (1) the PTSD prevalence rate in the HCWs (cut-off = 33) increased from 10.73% at T1 to 20.84% at T2, and the HCWs reported significantly higher PTSD scores at T2 than at T1 (*p* < 0.001); (2) risk perception was positively correlated with PTSD (*p* < 0.001); and (3) PTSD at T1 could significantly positively predict PTSD at T2 (*β* = 2.812, *p* < 0.01), and this longitudinal effect of PTSD at T1 on PTSD at T2 was mediated by risk perception at T2 (coefficient = 0.154, 95% CI = 0.023 to 0.297). Our data provide a snapshot of the worsening of HCWs’ PTSD along with the repeated pandemic outbreaks and highlight the important role of risk perception in the development of PTSD symptoms in HCWs over time.

## Introduction

On 11 March 2020, the World Health Organization (WHO) declared coronavirus disease 2019 (COVID-19) a pandemic outbreak due to the evolving nature of SARS-CoV-2 and the large number of global deaths. Despite efforts to end the global COVID-19 pandemic, subsequent outbreaks of the Delta variant (B.1.617.2) spread quickly and were identified as a more transmissible variant of SARS-CoV-2 [1]. In China, the first Delta variant outbreak was in Guangzhou in May 2021.

The repeated outbreaks of COVID-19 and its rapid transmission worldwide placed enormous pressure on the health care system and its professionals [2–6]. Health care workers in China faced great challenges during the severe pandemic, such as a wide knowledge gap of viral pathophysiology and the unprecedented local breakdown of protective equipment supplies [7]. These factors potentially aggravate stress, fear, and long-term negative mental health consequences in HCWs [8–11].

Several studies have shown that the outbreak of COVID-19 leads to high levels of PTSD in HCWs [12–16]. PTSD is a psychologically unbalanced state after experiencing or being exposed to traumatic events, which consists of symptoms specifically related to traumatic events, including intrusive re-experiencing, avoidance, negative alterations of cognition and mood, and excessive arousal or reactivity [17]. A survey-based study that enrolled 377 Chinese medical staff showed that the prevalence of PTSD was 3.8% in the early stage of the COVID-19 outbreak (approximately 1 month) [18]. A study by Johnson et al. (2020) revealed that 27.7% of 1,778 HCWs who had contact with patients during the COVID-19 outbreak showed clinical or subclinical PTSD symptoms [19].

Additionally, evidence suggests a close relationship between risk perception and PTSD. Risk perception refers to an individual’s intuitive evaluations of hazards in the environment to which they are exposed [20–22]. Risk perception differed among different individuals, which might affect their perceived risk levels of hazards and even their mental states, when they were exposed to traumatic events [23, 24]. Some pieces of evidence have found that risk perception could predict PTSD during the pandemic outbreak (e.g., SARS and COVID-19) [18, 25, 26].

Although a large number of cross-sectional studies have investigated the prevalence of PTSD in HCWs during the initial outbreak of COVID-19, the immediate PTSD levels in Chinese HCWs when confronted with the more transmissible Delta variant remain unclear. Along with the repeated outbreaks during the pandemic, the development of PTSD and risk perceptions in HCWs in a longitudinal manner is unknown. Additionally, although the close interrelation of risk perceptions and PTSD has been confirmed by some cross-sectional studies, there is still a lack of exploration about the role of risk perceptions in the evolution of PTSD symptoms with the repeated outbreaks during the pandemic.

To investigate the above issues, the present study aimed to (1) further examine the evolution of PTSD in HCWs after they successively encountered the initial and second waves of the COVID-19 pandemic and (2) further explore the role of risk perceptions in the evolution of PTSD over time. Accordingly, we conducted a one-year follow-up study to investigate PTSD in HCWs. The initial survey was carried out in the first wave of the pandemic (T1: May to June 2020), and the second survey was carried out during the pandemic outbreak caused by the Delta variant (T2: June 2021). The HCWs’ risk perceptions of COVID-19-related hazards were measured. We made three predictions: (1) given that a previous study suggested a trend of increased PTSD in COVID-19 survivors from 3 to 6 months after they left the hospital [27] and HCWs re-experienced traumatic events with the repeated outbreaks during the pandemic, we predicted that significantly more severe PTSD symptoms would be present in HCWs at the one-year follow-up; (2) in line with previous findings [18, 25, 26], we predicted a higher level of risk perception would be associated with more severe PTSD symptoms; and (3) we predicted PTSD in the early phase and risk perceptions might directly or indirectly result in the worsening of PTSD symptoms in the long term.

## Methods

### Study design and participants

The one-year follow-up study consisted of two sessions, which were performed at designated hospitals in Guangdong, China. Session 1 was conducted from May to June 2020 (T1). All participants provided their demographic information (age, sex, ethnic group, educational background, and classification of their medical profession) and completed a series of questionnaires, including the posttraumatic stress disorder checklist for DSM-5 (PCL-5) [28] and a self-reported risk perception questionnaire. Session 2 was carried out in June 2021 (T2) when an outbreak was caused by the Delta variant in Guangzhou, and HCWs in designated hospitals were invited to complete the same assessments as those in Session 1.

**Fig. 1:**
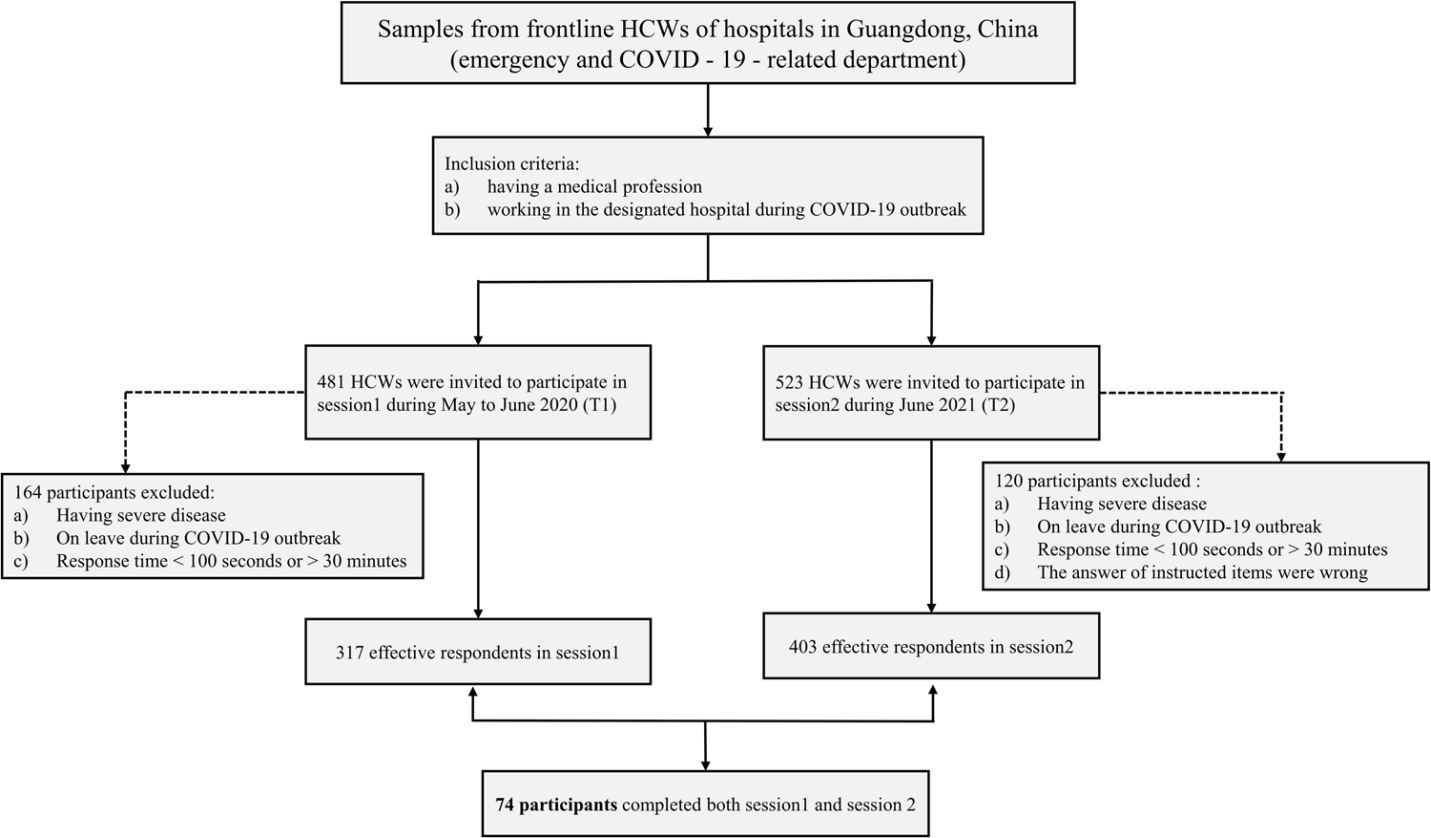
Sampling frame. Flowchart of the recruitment of frontline HCWs.

The sample size was calculated with *α* set as 0.05, *β* as 0.2, and the overall prevalence of PTSD estimated as 19.5% [16]. Based on the total number of HCWs in the designated hospitals, a minimum of 307 participants were required for this study. All HCWs who were medical professionals in the designated hospitals and working during the COVID-19 outbreak were eligible for participation. In total, for Session 1, online questionnaires were sent to 481 health care workers. Participants were excluded if they had severe disease (for example, acute enteritis, an upper respiratory infection with high fever and bone trauma), were on furlough during the period of the pandemic outbreak (more than 30 days), or had a response time <100 s or >30 min for completing the questionnaire. Finally, 317 participants completed Session 1 and were included in this study. In Session 2, 523 health care workers were invited to complete the survey, and the inclusion and exclusion criteria for the participants were the same as those in Session 1. Additionally, to identify and exclude the data provided by careless respondents, we used an attention check technique by including instructional items in the online questionnaires for Session 2 [29, 30]. Given that the respondents’ efforts might fluctuate when completing the questionnaire, we inserted two instructional items into the surveys, such as “Please select 2021 from the following numbers” and “Please indicate option [YES]” [31]. Finally, 403 participants completed Session 2 and were included for further analysis, and 74 HCWs participated in both sessions (see Fig. 1). The present study was approved by the Ethics Committee of Southern Medical University, and informed consent was obtained from all subjects.

### Measures

#### PTSD

The presence of PTSD in the HCWs was measured by the PTSD Checklist for DSM-5 (PCL-5). Twenty items were included in the PCL-5, and the participants were required to rate how bothered they had been in the previous month on a five-point Likert scale from 0 (not at all) to 4 (extremely). The summed score of all items defined the PTSD symptom severity, and there are four subscales for evaluating each relevant symptom: intrusion, avoidance, cognition/mood, and arousal/reactivity [28]. According to recent psychometric work, the cut-point of 33 seems to be sound for identifying probable significant PTSD symptom presentations. The Chinese version of the PCL-5 is widely employed by trauma-related studies and has been proven to be valid and have relatively high internal consistency [32]. In our dataset, the Cronbach’s alpha of the PCL-5 was 0.965 at T1 and T2.

#### Risk perception of COVID-19-related hazards

The risk perception questionnaire was formed based on the postulated psychometric model. Specific risk perception was measured for the dimensions of dread and familiarity [24]. These two dimensions were rated on a 5-point Likert scale for each hazard: from 1 (not dreadful at all) to 5 (extremely dreadful) regarding dread, as well as from 1 (extremely familiar) to 5 (not familiar at all) regarding familiarity. Four hazards were included based on previous studies: SAR-COV-2 virus (H1); COVID-19 disease (H2); COVID-19 patient or virus carrier (H3); and Treatment and prevention of COVID-19 (H4) [25, 33]. Accordingly, in our study, the self-rated questionnaire consisted of 8 items (2 dimensions × 4 hazards). A higher score indicates a higher level of a HCW’s risk perception of the pandemic. A recent study using factor analysis confirmed that H1 ~ H4 had an ideal model fit with sound reliability and validity [25]. In our dataset, Cronbach’s alpha for T1 and T2 was 0.836 and 0.873, respectively.

### Data analysis

All data were analysed using IBM SPSS Statistics and analysis of moment structures (AMOS). Descriptive analyses were conducted to examine the characteristics of the sample. The prevalence of PTSD was calculated by using a PCL-5 cut-off point of 33. The distribution of the PCL-5 scores and risk perceptions were examined. As a normal distribution of the data was not shown, two-tailed Mann–Whitney U tests were conducted to analyse the difference in the PCL-5 scores and risk perceptions between the two measurement time points (T1 vs. T2). Spearman correlations were applied to examine the relationships between PTSD and risk perception at both measurement time points.

Then, for the 74 frontline HCWs who participated in both sessions, hierarchical regression analysis was applied to explore potential predictors of PTSD at T2. Specifically, in the hierarchical regression models, PTSD at T2 was added as the dependent variable, and PTSD at T1, RP at T1, and RP at T2 were used as predictors. Notably, previous studies found that females, younger individuals, and those with a low level of education might have a higher risk of suffering from postpandemic PTSD, and working roles were also higher risk factors for developing PTSD [34]. Thus, in the hierarchical regression models, confounders including age, sex, ethnic group (Han, other minority), education level (senior high school or below, academy or bachelor’s, master’s or above) and classification of medical profession (doctor, nurse, other) were adjusted for (see Table 2). Additionally, we conducted mediation analyses to determine the potential mediating effect of risk perception at T2 on the relationship between PTSD at T1 and PTSD at T2 according to the results shown in the hierarchical multiple regression. First, we conducted bivariate correlations of the outcome variable (*Y* in the model, PTSD scores at T2), independent variable (*X* in the model, PTSD scores at T1) and mediation variable (*M* in the model, risk perceptions at T2) using Pearson correlation analysis. Second, by using mediation analysis in AMOS, we assessed how the relationship between PTSD scores at T1 and PTSD scores at T2 could be mediated by risk perceptions at T2 [35–37]. The 5,000 bootstrap method was applied to estimate the 95% bias-corrected CIs for the direct effect and indirect effect (two-tailed test, *α* = 0.05).

## Results

### Description of sample

In Session 1, the average age of the enrolled HCWs was 32 ± 8.93 years old, and 69.7% (*n* = 221) of the participants were women. The majority of the participants were of Han nationality (91.8%, *n* = 291). A total of 317 participants included 140 doctors (44.2%), 144 nurses (45.4%) and 33 other medical professionals (10.4%); 63.1% of the participants (*n* = 200) had university degrees, and 36.6% (*n* = 116) had master’s degrees or higher education backgrounds.

In Session 2, the data of 403 respondents were analysed (mean age = 32 ± 8.14 years old) and 269 of the respondents were women (66.7%). Most of the respondents were of Han nationality (95.3%, *n* = 384), 146 (36.2%) were doctors, 243 (60.3%) were nurses and 14 (3.5%) were other medical professionals; 74.9% of the participants (*n* = 302) had university degrees, and 23.5% (*n* = 95) had master’s degrees or higher education backgrounds (see Table S1).

### The differences in PTSD and risk perceptions between T1 and T2

**Fig. 2:**
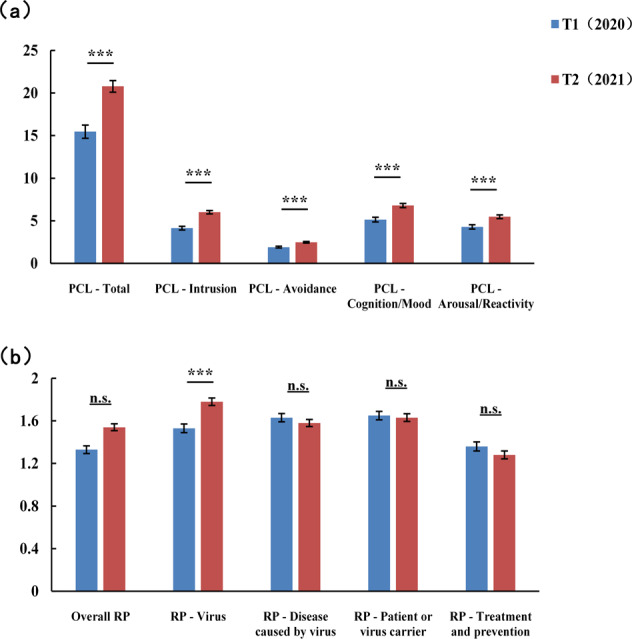
Differences in the main study variables between T1 and T2. **a** Comparison of the total scores of the PCL-5 and its four subscales between T1 and T2. **b** Comparison of the overall risk perception towards the pandemic and its four hazards between T1 and T2. PCL Posttraumatic Stress Disorder Checklist for DSM-5, RP risk perception towards the pandemic, T1 the first measurement point from May to June 2020, T2 the second measurement point from June 2021.

The PTSD prevalence rate increased from 10.73% at T1 to 20.84% at T2. As shown in Fig. 2a, the Mann–Whitney U tests revealed that the PCL-5 scores of the HCWs increased significantly from 15.47 (*SD* = 13.07) at T1 to 20.78 (*SD* = 14.27) at T2 (*Z* = −5.32, *p* < 0.001), and a significant increase at T2 relative to T1 was also observed for all four subscales of the PCL-50 (all *Z* ≥ 3.78, *p* < 0.001). For the 74 HCWs who completed the survey both at T1 and T2, the PCL-5 scores grew by ~27% from T1 to T2.

The overall risk perception towards the pandemic was 1.54 ± 0.69 (*M* ± *SD*) at T1 and 1.57 ± 0.62 at T2. The mean values for H1~H4 at T1 were 1.53 ± 0.74, 1.63 ± 0.72, 1.66 ± 0.74, and 1.36 ± 0.74, respectively. The mean values for H1–H4 at T2 were 1.78 ± 0.68, 1.58 ± 0.66, 1.63 ± 0.70, and 1.28 ± 0.74, respectively. As shown in Fig. 2b, the Mann–Whitney U tests showed no significant difference in overall risk perception towards the pandemic between T2 and T1 (*Z* = −0.73, *p* > 0.05), and the overall risk perception was estimated by the average value of the scores for the four hazards. The risk perception of the virus (H1) significantly increased from 1.53 at T1 to 1.78 at T2 (*Z* = −4.90, *p* < 0.001). However, risk perceptions of disease caused by the virus, a patient or a virus carrier, as well as treatment and prevention (H2–H4), showed no significant difference between T2 and T1, (all *p* > 0.05).

### The relationship between risk perceptions and PTSD at T1 and T2

Spearman correlation analysis results are shown in Table 1. The total scores of the PCL-5 and its four subscales were significantly positively associated with the overall risk perception towards the pandemic and its four hazards both at T1 and T2, suggesting a significantly close interrelationship between risk perception and PTSD at T1 and T2 (all *p* values were less than 0.001).

**Table 1:** Correlations between risk perception and PTSD in T1 and T2.

2020(T1)	1	2	3	4	5	6	7	8	9	10
PCL - Total	1.000									
PCL - Intrusion	0.904***	1.000								
PCL - Avoidance	0.785***	0.743***	1							
PCL - Cognition/Mood	0.931***	0.766***	0.657***	1						
PCL - Arousal/Reactivity	0.916***	0.764***	0.596***	0.845***	1					
Overall RP	0.290***	0.309***	0.223***	0.284***	0.247***	1				
RP - Virus	0.292***	0.303***	0.209***	0.290***	0.256***	0.931***	1			
RP - Disease caused by virus	0.256***	0.261***	0.194***	0.257***	0.221***	0.940***	0.860***	1		
RP - Patient or virus carrier	0.240***	0.243***	0.185***	0.254***	0.193***	0.934***	0.837***	0.881***	1	
RP - Treatment and prevention	0.259***	0.306***	0.214***	0.237***	0.219***	0.875***	0.742***	0.743***	0.751***	1
2021(T2)	1	2	3	4	5	6	7	8	9	10
PCL - Total	1.000									
PCL - Intrusion	0.885***	1								
PCL - Avoidance	0.805***	0.776***	1							
PCL - Cognition/Mood	0.932***	0.724***	0.671***	1						
PCL - Arousal/Reactivity	0.911***	0.708***	0.598***	0.851***	1					
Overall RP	0.332***	0.353***	0.255***	0.298***	0.286***	1				
RP - Virus	0.290***	0.348***	0.239***	0.224***	0.248***	0.858***	1			
RP - Disease caused by virus	0.318***	0.333***	0.259***	0.284***	0.273***	0.934***	0.824***	1		
RP - Patient or virus carrier	0.270***	0.290***	0.222***	0.239***	0.233***	0.910***	0.757***	0.877***	1	
RP - Treatment and prevention	0.316***	0.281***	0.210***	0.326***	0.291***	0.809***	0.522***	0.649***	0.624***	1

*p value less than 0.05, **p value less than 0.01, ***p value less than 0.001.

### Longitudinal effects of PTSD at T1 on PTSD at T2 and the mediating role of risk perception at T2

#### Hierarchical regression analysis

For the 74 HCWs who participated in both sessions (for characteristics of the sample, see Table S2), we used hierarchical regression analysis to further explore the longitudinal effects of risk perception and PTSD at T1 on PTSD at T2. The results are shown in Table 2. No significant effect of the demographic variables on PTSD at T2 was observed in Model 1. In Model 2, PTSD at T1 (*β* = 2.81, *p* < 0.01) and RP at T2 (*β* = 6.13, *p* < 0.001) were significantly correlated with the PTSD score at T2.

**Table 2:** Regression analysis with the total score of PCL-5 of HCWs in T2 as the outcome variable.

Model		PCL - Total (T2)					Adjusted R^2^	ΔR^2^	ΔF
		Beta	SE	*β*	*t*	R^2^			
**1**	(Constant)	15.945	22.519		0.708	0.070	−0.014	0.070	0.838
	Sex	8.887	5.319	0.245	1.671				
	Age	−0.277	0.263	−0.134	−1.052				
	Ethnic	4.647	7.020	0.080	0.662				
	Nurse versus other	−12.473	15.109	−0.334	−0.826				
	Doctor versus other	−20.098	16.902	−0.524	−1.189				
	Education	6.303	6.300	0.164	1.000				
**2**	(Constant)	−4.591	17.144		−0.268	0.510	0.441	0.440	19.170***
	Sex	5.072	4.041	0.140	1.255				
	Age	−0.174	0.199	−0.084	−0.874				
	Ethnic	−1.808	5.304	−0.031	−0.341				
	Nurse versus other	−4.314	11.273	−0.116	−0.383				
	Doctor versus other	−7.853	12.687	−0.205	−0.619				
	Education	5.629	4.684	0.146	1.202				
	RP-total (T1)	−1.339	1.808	−0.069	−0.741				
	RP-total (T2)	11.754	1.918	0.578	6.128***				
	PCL-total (T1)	0.311	0.111	0.264	2.812**				

*В* Unstandardized beta, *β* Standardized regression weight.

*p value less than 0.05, **p value less than 0.01, ***p value less than 0.001.

#### Mediation analysis

The Spearman correlation analysis of the observed variables in mediation analysis revealed that PTSD at T1 was significantly positively associated with PTSD at T2 (*r* = 0.477, *p* < 0.001) and RP at T1 (*r* = 0.271, *p* = 0.020), and RP at T2 was significantly positively correlated with PTSD at T2 (*r* = 0.619, *p* < 0.001).

**Fig. 3:**
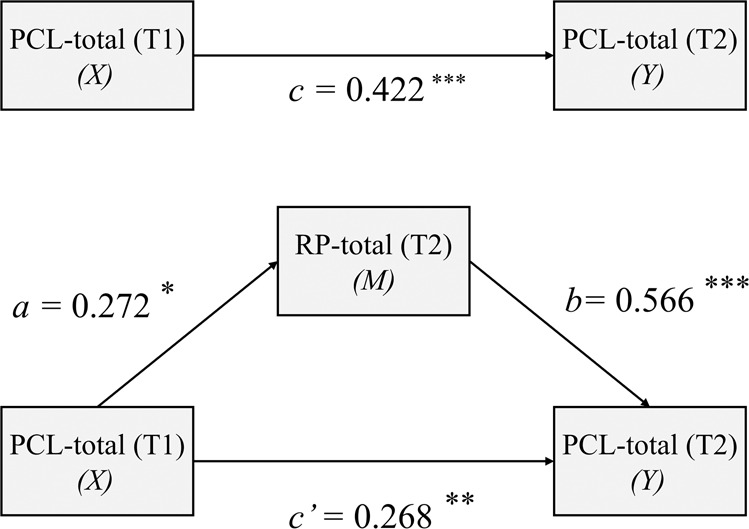
Risk perception at T2 mediates the relationship between PTSD at T1 and PTSD at T2. *p value less than 0.05, **p value less than 0.01, ***p value less than 0.001.

Mediation analysis showed that PTSD at T1 could positively predict risk perception at T2 (coefficient = 0.272, *p* = 0.016, path *a* in Fig. 3), and risk perception at T2 predicted PTSD at T2 (coefficient = 0.566, *p* < 0.001, path *b* in Fig. 3). Zero was not included in a bias-corrected bootstrap-confidence interval (CI), indicating that the indirect effect was significant. Hence, the indirect effect from PTSD at T1 to PTSD at T2 through RP at T2 was significant (coefficient = 0.154, SE = 0.069, 95% CI = 0.023 to 0.297). Additionally, in this model, the direct effect from PTSD at T1 to PTSD at T2 remained significant (coefficient = 0.268, *p* = 0.002, path *c’* in Fig. 3), indicating that RP at T2 partially mediated the relationship between PTSD at T1 and PTSD at T2.

## Discussion

In the present one-year longitudinal follow-up study, we explored the development and relationship of PTSD symptoms and risk perceptions of HCWs during two COVID-19 outbreaks. The results suggested that (1) the PTSD prevalence rate in health care workers (cut-off = 33) at T1 and T2 was 10.73% and 20.84%, respectively, and the HCWs reported significantly more severe PTSD in the second wave of COVID-19 (Delta outbreak); (2) the overall risk perception of COVID-19-related hazards remained relatively stable from T1 to T2 and was positively associated with PTSD severity both at T1 and T2; and (3) PTSD at T1 significantly positively predicted PTSD at T2, and risk perception at T2 mediated this increase in PTSD from T1 to T2.

A recent meta-analysis and systematic review revealed that the prevalence rate of PTSD during COVID-19 pandemic outbreaks was 9%, including 11 studies conducted from February to May 2020 [15]. This rate was similar to the prevalence rate of HCWs at T1 (May to June 2020) in the present study (10.73%). It is worth noting that the prevalence rate at T2 in the present study (20.84%) was double the rate reported at T1. The high prevalence rates in the HCWs reflected that they were at high risk of repeated traumatic stress and were a population that was vulnerable to PTSD. Meanwhile, our analysis revealed that the total scores of the PCL-5 at T2 were significantly higher than those at T1, and for the HCWs who completed the survey at both T1 and T2, the PCL-5 scores grew by ~27% from T1 to T2, indicating that PTSD has become an increasingly serious problem in health care workers with the repeated outbreaks during the pandemic. Similarly, a study from COVID-19 survivors in Wuhan also showed a trend of increasing PTSD, and the results revealed that COVID-19 survivors’ PCL-5 scores grew by ~20% from 3 to 6 months after they left the hospital [27]. In contrast, a recent longitudinal study that focused on the mental states of the general public in China during the COVID-19 pandemic suggested that posttraumatic stress symptoms decreased from 1 to 3 months after the pandemic outbreak [38]. These results suggested that with the substantially increasing numbers of recovered COVID-19 patients, the mental states of the general public gradually improved. However, compared to the general public, HCWs and COVID-19 survivors might be more traumatized during the pandemic; therefore, it might be harder and take longer for them to recover from PTSD. Furthermore, the regression analysis showed that the overall PTSD level at T1 could significantly positively predict the overall PTSD level at T2, and further analysis of the subscales of the PCL-5 is shown in Tables S3–S6 in the Supplementary material. These results demonstrated the persistence and even worsening of PTSD symptoms for a substantial proportion of HCWs at the one-year follow-up. This is in accordance with previous epidemiological findings that the more exposure to multiple traumatic events, the greater the risk of suffering from PTSD [39–43]. Notably, our longitudinal data suggested that HCWs might develop worse PTSD without intervention at the early stage. Since the COVID-19 pandemic is still ongoing and outbreaks are occurring repeatedly, HCWs’ mental health problems and the steady worsening of their PTSD symptoms over time need urgent attention.

For risk perception, we explored the evolution of risk perception over a period of one year, and the results suggested that the overall risk perception towards the pandemic remained relatively stable from T1 to T2. In line with previous studies, in two successive outbreaks of the pandemic, the risk perception of hazards during the COVID-19 pandemic was associated with PTSD severity, with those who had significantly higher risk perceptions for COVID-19-related hazards reporting more severe PTSD symptoms [20, 25, 44]. These results suggested the important role of COVID-19-related risk perceptions in HCWs’ PTSD symptoms.

Beyond these findings, our dataset showed the mediating role of risk perception at T2 on the increase in PTSD with two pandemic outbreaks. Further structural equation modelling that considered the subscales of the PCL-5 and four hazards of risk perceptions also showed consistent results (see Figs. S1–S2 in the Supplementary material). Specifically, those who had a higher level of PTSD at T1 seemed to report a higher risk perception of COVID-19-related hazards at T2, which resulted in higher PTSD scores at T2. Previous studies have reported that PTSD, a mental disease that develops after people are exposed to frightening and even life-threatening traumatic events, might cause long-term persistent negative alterations in cognition and mood [18, 45–47]. Accordingly, the present findings suggested the possibility that HCWs with more severe PTSD at T1 suffered from more negative cognition and mood over an extended period, and these long-term persistent psychological consequences of PTSD might have increased their intuitive risk perception of hazards at T2, which further increased their vulnerability to future traumatic events and led to a higher level of PTSD at T2. Such findings provide preliminary evidence that higher risk perception might be an important contributor to the increase in PTSD symptoms at T2 relative to T1. In addition, it seemed that the association of more severe PTSD symptoms at the early stage with the subsequent higher risk perception might comprise a significant predictor of worse PTSD symptoms over time. Furthermore, improving the capacity of risk resistance by adjusting risk perception could be an effective intervention target for preventing the worsening of PTSD, such as health education, especially for HCWs with severe PTSD in their first contact with the pandemic.

## Limitations

The findings in this study should be interpreted with caution due to several limitations. First, we only recruited HCWs for both sessions from Guangzhou, and studies on HCWs from other pandemic cities are essential for validation. Second, only 74 HCWs took part in both surveys due to strict quarantine standards and work overload. To generalize and to obtain more precise findings, we need larger-scale data to better investigate the predictors and dynamic development of PTSD symptoms in HCWs over time.

## Conclusion

Our data provide a snapshot that HCWs’ PTSD symptoms were aggravated with the repeated outbreaks of COVID-19 within a one-year period. Among HCWs from designated hospitals, over 10.73% suffered from PTSD in the first wave of the COVID-19 pandemic, and the prevalence rate increased dramatically to 20.84% in the second wave of the pandemic. Furthermore, our longitudinal data revealed the mediating role of risk perception in the increase in PTSD over time. The present study highlighted the necessity of early intervention to prevent the worsening of PTSD in HCWs over time and suggested that improving the capacity of adjusting risk perceptions could be an effective intervention target.

## Supplementary information

Supplementary material

Fig.S1

Fig.S2
