# Supplementary material for: The increase of PTSD in front-line health care workers during the COVID-19 pandemic and the mediating role of risk perception: a one-year follow-up study
Source: Transl Psychiatry. 2022 May 3;12:180. doi: 10.1038/s41398-022-01953-7 (PMC9062850; doi:10.1038/s41398-022-01953-7)
Supplement: Supplementary file 1 — Supplementary material [file 41398_2022_1953_MOESM1_ESM.docx]

**Table S1 Characteristics of the sample in T1 and T2**

|  |  | **T1 (N=317)** | |  | **T2 (N=403)** | |
| --- | --- | --- | --- | --- | --- | --- |
|  |  | Samples | Percentage |  | Samples | Percentage |
| **Age** | |  |  |  |  |  |
|  | 20-30 years old | 163 | 51.4 |  | 211 | 52.4 |
|  | 31-40 years old | 101 | 31.9 |  | 139 | 34.5 |
|  | Above 40 years old | 53 | 16.7 |  | 56 | 13.9 |
| **Gender** | |  |  |  |  |  |
|  | Male | 96 | 30.3 |  | 134 | 33.3 |
|  | Female | 221 | 69.7 |  | 269 | 66.7 |
| **Ethnic group** | |  |  |  |  |  |
|  | Han | 291 | 91.8 |  | 384 | 95.3 |
|  | Minorities | 26 | 8.2 |  | 19 | 4.7 |
| **Education Level** | |  |  |  |  |  |
|  | Senior high school or below | 1 | 0.3 |  | 6 | 1.5 |
|  | Academy or bachelor | 200 | 63.1 |  | 302 | 74.9 |
|  | Master or above | 116 | 36.6 |  | 95 | 23.5 |
| **Classification of medical profession** | |  |  |  |  |  |
|  | Doctor | 140 | 44.2 |  | 146 | 36.2 |
|  | Nurse | 144 | 45.4 |  | 243 | 60.3 |
|  | Others | 33 | 10.4 |  | 14 | 3.5 |

**Table S2 Characteristics of the HCWs participant in both session 1 and session 2**

| N=74 | | | |
| --- | --- | --- | --- |
|  |  | Samples | Percentage |
| Age | |  |  |
|  | 20-30 years old | 36 | 48.6 |
|  | 31-40 years old | 32 | 43.2 |
|  | Above 40 years old | 6 | 8.1 |
| Gender | |  |  |
|  | Male | 15 | 20.3 |
|  | Female | 59 | 79.7 |
| Ethnic group | |  |  |
|  | Han | 69 | 93.2 |
|  | Minorities | 5 | 6.8 |
| Education Level | |  |  |
|  | Senior high school or below | 0 | 0 |
|  | Academy or bachelor | 65 | 87.8 |
|  | Master or above | 9 | 12.2 |
| Classification of medical profession | |  |  |
|  | Doctor | 13 | 17.6 |
|  | Nurse | 60 | 81.1 |
|  | Others | 1 | 1.4 |

**Table S3 Regression analysis with the total score of PCL - Criterion B of HCWs in T2 as the outcome variable**

|  |  | **PCL - Criterion B (T2)** | | | | |  |  |  |
| --- | --- | --- | --- | --- | --- | --- | --- | --- | --- |
| **Model** |  | **Beta** | **SE** | ***β*** | ***t*** | **R^2^** | **Adjusted R^2^** | **ΔR^2^** | **ΔF** |
| **1** | (Constant) | 1.624 | 6.576 |  | 0.247 | 0.066 | -0.017 | .066 | 0.793 |
|  | Sex | 2.168 | 1.553 | 0.205 | 1.396 |  |  |  |  |
|  | age | -0.034 | 0.077 | -0.057 | -0.448 |  |  |  |  |
|  | Ethnic | 2.407 | 2.050 | 0.142 | 1.174 |  |  |  |  |
|  | Nurse versus other | -3.787 | 4.412 | -0.348 | -0.858 |  |  |  |  |
|  | Doctor versus other | -6.509 | 4.935 | -0.582 | -1.319 |  |  |  |  |
|  | Education | 2.279 | 1.840 | 0.203 | 1.239 |  |  |  |  |
| **2** | (Constant) | -2.454 | 5.770 |  | -0.425 | .349 | .257 | .283 | 9.261^***^ |
|  | Sex | 1.380 | 1.358 | 0.130 | 1.016 |  |  |  |  |
|  | age | -0.021 | 0.067 | -0.035 | -0.319 |  |  |  |  |
|  | Ethnic | 0.740 | 1.791 | 0.044 | 0.413 |  |  |  |  |
|  | Nurse versus other | -1.761 | 3.800 | -0.162 | -0.463 |  |  |  |  |
|  | Doctor versus other | -3.698 | 4.279 | -0.331 | -0.864 |  |  |  |  |
|  | Education | 2.197 | 1.573 | 0.196 | 1.396 |  |  |  |  |
|  | RP-total (T1) | -0.699 | 0.610 | -0.123 | -1.145 |  |  |  |  |
|  | RP-total (T2) | 3.142 | 0.629 | 0.530 | 4.994^***^ |  |  |  |  |
|  | PCL-Criterion B (T1) | 0.150 | 0.118 | 0.136 | 1.273 |  |  |  |  |

**Table S4 Regression analysis with the total score of PCL - Criterion C of HCWs in T2 as the outcome variable**

|  |  | **PCL - Criterion C (T2)** | | | | |  |  |  |
| --- | --- | --- | --- | --- | --- | --- | --- | --- | --- |
| **Model** |  | **Beta** | **SE** | ***β*** | ***t*** | **R^2^** | **Adjusted R^2^** | **ΔR^2^** | **ΔF** |
| **1** | (Constant) | 0.359 | 2.902 |  | 0.124 | 0.071 | -0.012 | 0.071 | 0.851 |
|  | Sex | 0.767 | 0.686 | 0.164 | 1.118 |  |  |  |  |
|  | age | 0.001 | 0.034 | 0.004 | 0.028 |  |  |  |  |
|  | Ethnic | -0.219 | 0.905 | -0.029 | -0.242 |  |  |  |  |
|  | Nurse versus other | -1.392 | 1.947 | -0.290 | -0.715 |  |  |  |  |
|  | Doctor versus other | -3.223 | 2.178 | -0.651 | -1.480 |  |  |  |  |
|  | Education | 1.536 | 0.812 | 0.309 | 1.892 |  |  |  |  |
| **2** | (Constant) | -1.225 | 2.593 |  | -0.473 | 0.323 | 0.228 | 0.252 | 7.943^***^ |
|  | Sex | 0.605 | 0.613 | 0.129 | 0.987 |  |  |  |  |
|  | age | -0.003 | 0.030 | -0.013 | -0.114 |  |  |  |  |
|  | Ethnic | -0.884 | 0.804 | -0.118 | -1.100 |  |  |  |  |
|  | Nurse versus other | -0.270 | 1.719 | -0.056 | -0.157 |  |  |  |  |
|  | Doctor versus other | -1.840 | 1.933 | -0.372 | -0.952 |  |  |  |  |
|  | Education | 1.450 | 0.710 | 0.292 | 2.043 |  |  |  |  |
|  | RP-total (T1) | -0.359 | 0.274 | -0.143 | -1.309 |  |  |  |  |
|  | RP-total (T2) | 0.953 | 0.289 | 0.363 | 3.299^***^ |  |  |  |  |
|  | PCL-Criterion C (T1) | 0.350 | 0.122 | 0.309 | 2.860^***^ |  |  |  |  |

**Table S5 Regression analysis with the total score of PCL - Criterion D of HCWs in T2 as the outcome variable**

|  |  | **PCL - Criterion D (T2)** | | | | |  |  |  |
| --- | --- | --- | --- | --- | --- | --- | --- | --- | --- |
| **Model** |  | **Beta** | **SE** | ***β*** | ***t*** | **R^2^** | **Adjusted R^2^** | **ΔR^2^** | **ΔF** |
| **1** | (Constant) | 8.046 | 8.536 |  | 0.943 | 0.080 | -0.003 | 0.080 | 0.968 |
|  | Sex | 3.323 | 2.016 | 0.240 | 1.648 |  |  |  |  |
|  | age | -0.131 | 0.100 | -0.166 | -1.313 |  |  |  |  |
|  | Ethnic | 0.751 | 2.661 | 0.034 | 0.282 |  |  |  |  |
|  | Nurse versus other | -5.554 | 5.727 | -0.391 | -0.970 |  |  |  |  |
|  | Doctor versus other | -7.922 | 6.407 | -0.542 | -1.236 |  |  |  |  |
|  | Education | 2.380 | 2.388 | 0.162 | 0.996 |  |  |  |  |
| **2** | (Constant) | -0.525 | 6.078 |  | -0.086 | 0.575 | 0.515 | 0.495 | 24.868^***^ |
|  | Sex | 1.687 | 1.434 | 0.122 | 1.176 |  |  |  |  |
|  | age | -0.087 | 0.070 | -0.110 | -1.232 |  |  |  |  |
|  | Ethnic | -1.685 | 1.880 | -0.076 | -0.896 |  |  |  |  |
|  | Nurse versus other | -2.812 | 3.996 | -0.198 | -0.704 |  |  |  |  |
|  | Doctor versus other | -3.460 | 4.495 | -0.237 | -0.770 |  |  |  |  |
|  | Education | 2.190 | 1.661 | 0.149 | 1.318 |  |  |  |  |
|  | RP-total (T1) | -0.120 | 0.638 | -0.016 | -0.188 |  |  |  |  |
|  | RP-total (T2) | 4.436 | 0.683 | 0.572 | 6.498^***^ |  |  |  |  |
|  | PCL-Criterion D (T1) | 0.423 | 0.114 | 0.321 | 3.726^***^ |  |  |  |  |

**Table S6 Regression analysis with the total score of PCL - Criterion E of HCWs in T2 as the outcome variable**

|  |  | **PCL - Criterion E (T2)** | | | | |  |  |  |
| --- | --- | --- | --- | --- | --- | --- | --- | --- | --- |
| **Model** |  | **Beta** | **SE** | ***β*** | ***t*** | **R^2^** | **Adjusted R^2^** | **ΔR^2^** | **ΔF** |
| **1** | (Constant) | 5.915 | 6.953 |  | 0.851 | 0.066 | -0.018 | 0.066 | 0.783 |
|  | Sex | 2.629 | 1.642 | 0.235 | 1.601 |  |  |  |  |
|  | age | -0.112 | 0.081 | -0.176 | -1.381 |  |  |  |  |
|  | Ethnic | 1.709 | 2.167 | 0.095 | 0.788 |  |  |  |  |
|  | Nurse versus other | -1.740 | 4.665 | -0.152 | -0.373 |  |  |  |  |
|  | Doctor versus other | -2.443 | 5.218 | -0.207 | -0.468 |  |  |  |  |
|  | Education | 0.107 | 1.945 | 0.009 | 0.055 |  |  |  |  |
| **2** | (Constant) | -0.361 | 5.359 |  | -0.067 | 0.493 | 0.422 | 0.427 | 17.981^***^ |
|  | Sex | 1.397 | 1.266 | 0.125 | 1.103 |  |  |  |  |
|  | age | -0.056 | 0.063 | -0.088 | -0.896 |  |  |  |  |
|  | Ethnic | 0.170 | 1.657 | 0.010 | 0.103 |  |  |  |  |
|  | Nurse versus other | 0.154 | 3.528 | 0.013 | 0.044 |  |  |  |  |
|  | Doctor versus other | 0.728 | 3.969 | 0.062 | 0.183 |  |  |  |  |
|  | Education | -0.235 | 1.469 | -0.020 | -0.160 |  |  |  |  |
|  | RP-total (T1) | -0.120 | 0.566 | -0.020 | -0.211 |  |  |  |  |
|  | RP-total (T2) | 2.978 | 0.611 | 0.475 | 4.875^***^ |  |  |  |  |
|  | PCL-Criterion E (T1) | 0.394 | 0.108 | 0.355 | 3.652^***^ |  |  |  |  |

**Fig. S1 Structural equation model of the mediation role of risk perception in the increasement of PTSD in HCWs.** * = *p* value less than 0.05; ** = *p* value less than 0.01; *** = *p* value less than 0.001.

**Fig. S2 Structural equation model of the mediation role of each hazard in the increasement of PTSD in HCWs.** * = *p* value less than 0.05; ** = *p* value less than 0.01; *** = *p* value less than 0.001, bold coefficient and CI means the indirect effect in the model was significant.
