# Supplementary figures and images for: The increase of PTSD in front-line health care workers during the COVID-19 pandemic and the mediating role of risk perception: a one-year follow-up study
Source: Transl Psychiatry. 2022 May 3;12:180. doi: 10.1038/s41398-022-01953-7 (PMC9062850; doi:10.1038/s41398-022-01953-7)

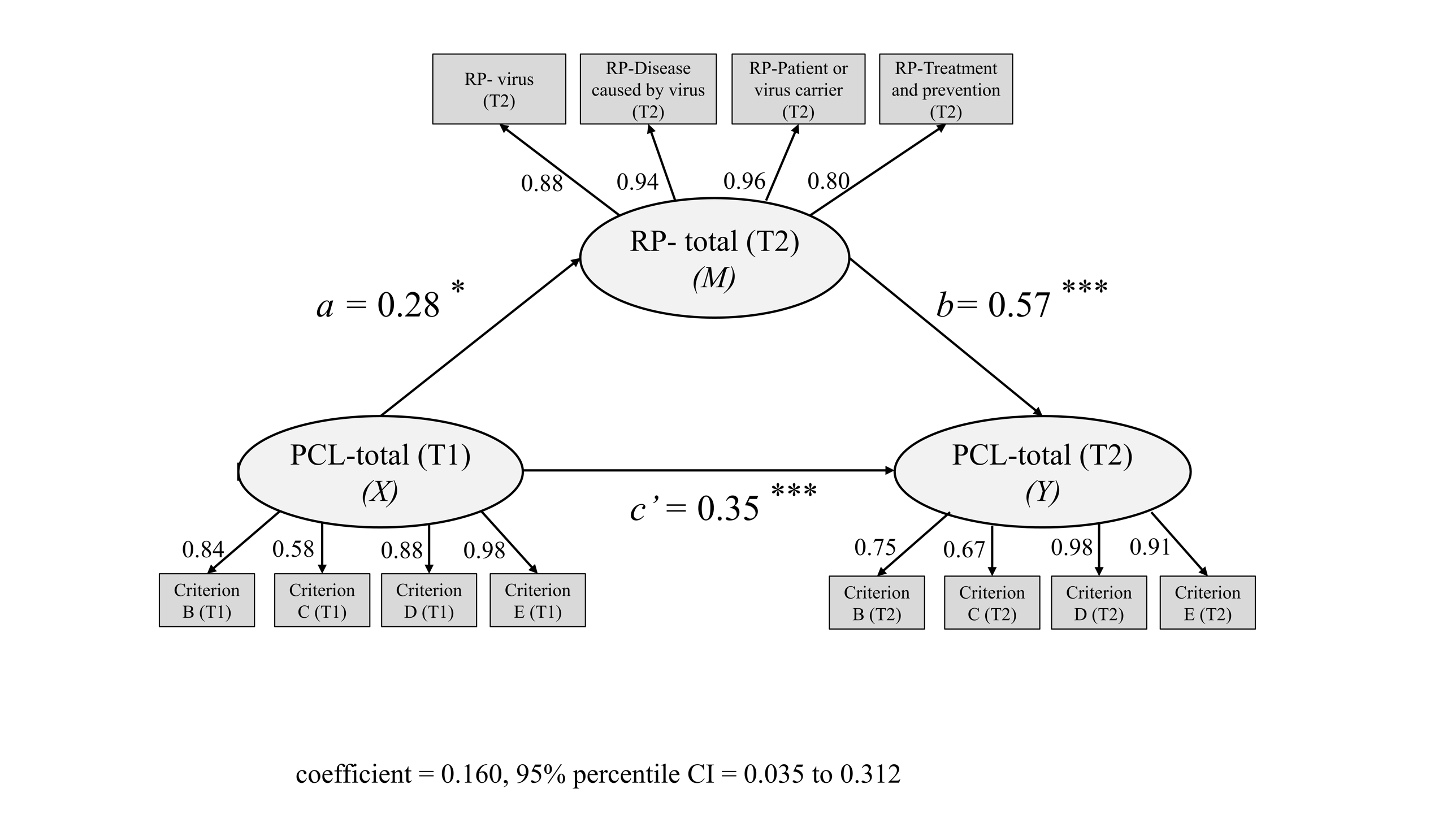

Supplement: Supplementary file 2 — Fig.S1 [file 41398_2022_1953_MOESM2_ESM.tif]

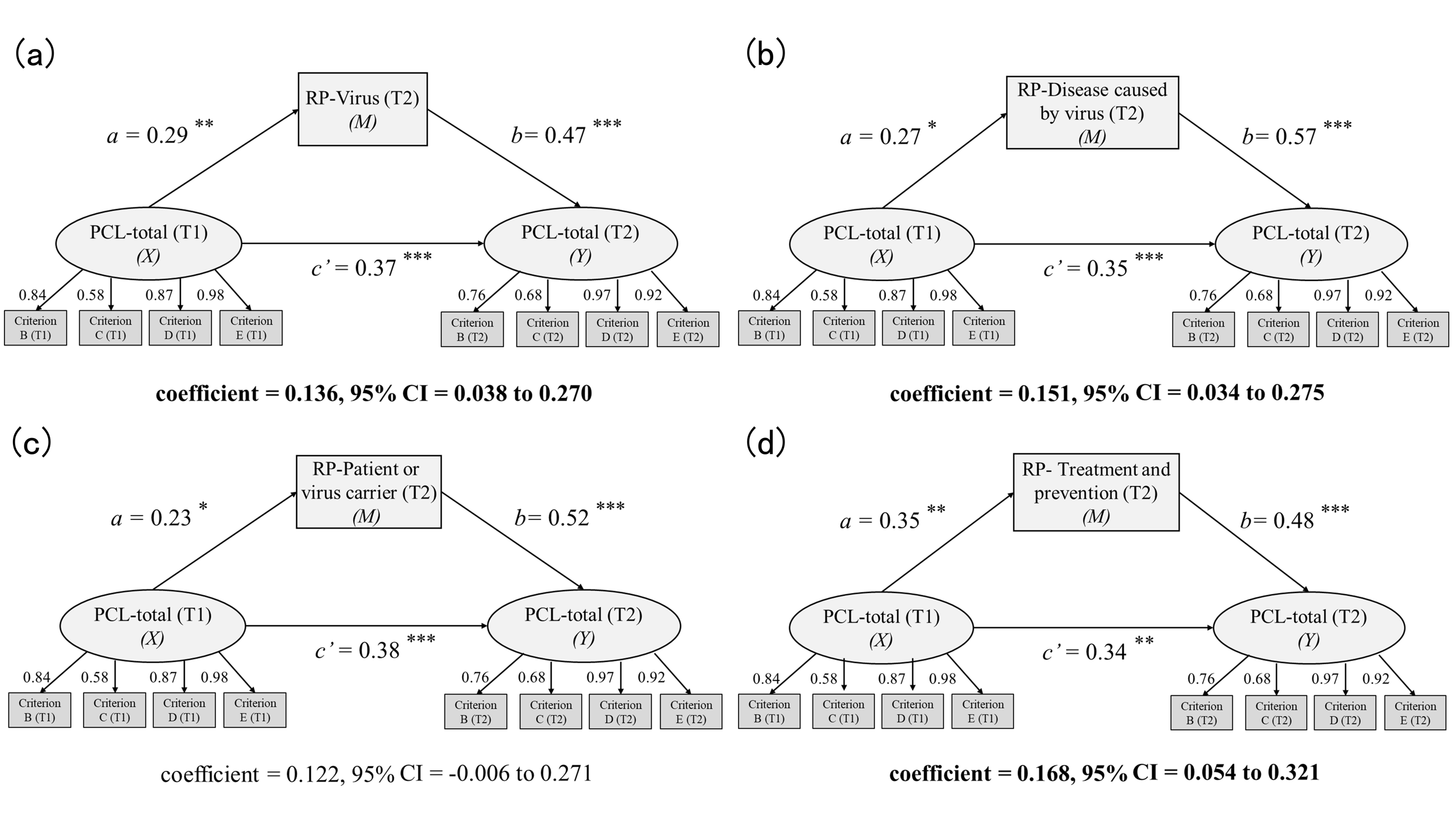

Supplement: Supplementary file 3 — Fig.S2 [file 41398_2022_1953_MOESM3_ESM.tif]
